# Dosimetry and treatment planning of Occu-Prosta I-125 seeds for intraocular lesions

**DOI:** 10.4103/0971-6203.39419

**Published:** 2008

**Authors:** Suresh Chaudhari, Sudesh Deshpande, Vivek Anand, Sandeep De, Sanjay Saxena, A. Dash, Mahua Basu, Preetam Samant, V. Kannan

**Affiliations:** Department of Radiation Oncology, P. D. Hinduja National Hospital and MRC, Mumbai, India; 1 Department of Radiopharmaceutical Division, BARC, Mumbai, India; 2 Department of Radiological Physics and Advisory Division, BARC, Mumbai, India; 3 Department of Ophthalmology, P. D. Hinduja National Hospital and MRC, Mumbai, India

**Keywords:** I-125, dosimetry and planning, eye plaque, intraocular lesions

## Abstract

Intraocular malignant lesions are frequently encountered in clinical practice. Plaque brachytherapy represents an effective means of treatment for intraocular lesions. Recently Radiopharmaceutical Division, BARC, Mumbai, has indigenously fabricated reasonable-cost I-125 sources. Here we are presenting the preliminary experience of dosimetry of sources, configuration of treatment planning system (TPS) and quality assurance (QA) for eye plaque therapy with Occu-Prosta I-125 seeds, treated in our hospital, for a patient with ocular lesions. I-125 seeds were calibrated using well-type chamber. BrachyVision TPS was configured with Monte Carlo computed radial dose functions and anisotropy functions for I-125 sources. Dose calculated by TPS at different points in central axis and off axis was compared with manually calculated dose. Eye plaque was fabricated of 17 karat pure gold, locally. The seeds were arranged in an outer ring near the edge of the plaque and in concentric rings throughout the plaque. The sources were manually digitized on the TPS, and dose distribution was calculated in three dimensions. Measured activity using cross-calibrated well-type chamber was within ±10% of the activity specified by the supplier. Difference in TPS-calculated dose and manually calculated dose was within 5%. Treatment time calculated by TPS was in concordance with published data for similar plaque arrangement.

Intraocular malignant lesions, primary or metastatic, are frequently encountered in clinical practice, and the common treatment decision has been enucleation.[1] Literature studies reveal globe preservation with nonsurgical treatment, using radiation therapy with possible preservation of vision. The dose of teleradiation with megavoltage therapy required to control the primary tumor like melanomas or metastasis can exceed the tolerance of retina, optic nerve, lens, eyelids and lashes. The treatment plan and choice of radiation delivery system must optimize dose distribution to minimize treatment morbidity. Alternative to fractionated megavoltage teleradiation, charged particles such as proton or helium ions, radiosurgery or brachytherapy has been attempted. Charl *et al.* have shown that survival results of eye plaque brachytherapy are similar to charged-particle therapy for treatment of uveal melanoma.[2] Hence plaque brachytherapy represents a practical alternative for treatment of intraocular lesions.

Eye plaques can be of permanent-loading type or temporary loaded. Historically Ra-226 and Co-60 were used for treatment. The isotopes in use at present are 90-Sr/Yr-90, 106-Ru/Rh-106 and I-125. Except I-125, all are permanently loaded. I-125 has gained popularity in recent years due to development of miniature source and its easy availability.

American Brachytherapy Society (ABS) and Collaborative Ocular Melanoma Study (COMS) have developed guidelines for I-125 eye plaque therapy.[3–5] With standard source configuration, eye plaques are commercially available. The high cost of these commercially available sources has been overcome by indigenously fabricated reasonable-cost I-125 sources by Radiopharmaceutical Division of Bhabha Atomic Research Centre (BARC), Mumbai, with trade name Occu-Prosta. This development has opened the gateway for affordable eye plaque treatment.

Because of steep dose gradient, high spatial resolution, tumor dimension and location, eye plaque therapy becomes a challenging job for medical physicist and radiation oncologist.

Here we describe the preliminary experience of dosimetry of sources, configuration of treatment planning system (TPS) and quality assurance (QA) for eye plaque therapy with I-125 for a patient with ocular metastasis treated in our hospital.

## Materials and Methods

### I-125 sources

**Figure 1:**
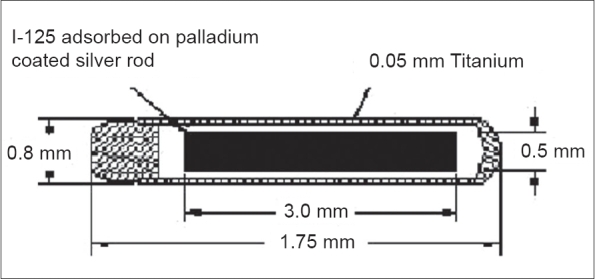
Sectional diagram of Occu-Prosta seed

I-125 Occu-Prosta seed of BARC consists of I-125 adsorbed on 0.5 mm(Φ) × 3 mm(L) palladium-coated silver rod with seed strength up to 4.45U (1U = 1 cGy cm^2^ h^−1^). The radiation source is laser welded in a tiny titanium capsule of 0.8 mm(Φ) × 4.75 mm(L) [Figure 1]. Titanium encapsulation, in addition to being inert towards source matrix, assures good tissue compatibility and together with the palladium-coated silver rod results in a total self-absorption of approximately 35% gamma photons. I-125 decays with half life of 59.4 days by electron capture with the emission of auger electrons, characteristic 27.4 and 31.4 keV X-rays (∼140%) and 35.5 keV gamma photons (∼7%). The electrons are however absorbed by the titanium wall of the capsule and do not interfere in the dosimetric considerations. Fluorescent X-rays of 22.1 and 25.2 keV resulting from silver rods (i.e., the source core) are also emitted. The sources are leak tested and are free from surface contamination.

### Calibration of sources

I-125 Occu-Prosta seeds were supplied with calibration certificate. The source strength was specified in mCi and MBq. The individual seeds were also specified in terms of air kerma rate, within ± 10%. Prior to using newly received sources for treatment, the vendor-supplied calibrations must be verified.[6–8] Well-type ion chamber has been recommended by ICRU for specification of brachytherapy sources.[6–9] On the receipt of sources, the activity was checked with re-entrant well-type ion chamber. The chamber used was 1000-cc well-type chamber, and the electrometer was CDX2000 (Standard Imaging, Middleton, Wisconsin, USA). The chamber was cross-calibrated with the secondary standard re-entrant ion chamber from BARC for I-125.

For the activity calibration, special jig was used. The jig consists of a plastic tube which can hold the source with 5-mm scale marked on it. By manually moving the source in this tube, the plateau and peak response were found. The voltage applied was 300 V, and current was measured for 300 s.

As per TG56 guideline, a minimum 10% of loose sources should be calibrated by the end user. Six sources out of total 15 sources were randomly checked.

### Dosimetry data of seeds

The dosimetric studies for Occu-Prosta seeds have been done by at least two investigators.[10,11] The study involved absolute dose rate measurements and Monte-Carlo simulation, which includes calculation of the dose rate constant, i.e., the dose rate at a distance of 1 cm per unit reference air kerma rate. The dosimetric studies were compared with TLD measurements and were found to be in concordance. These two studies were used as a baseline for dose calculation.

According to TG43 protocol, dose distribution from a line source in two dimensions can be described in terms of a polar coordinate system with its origin at the source center, where r is the distance to the point of interest and θ is the angle with respect to the long axis of the source. The dose rate D (r, θ) at a point (r, θ) is given as

$D(r,\theta )=\Lambda S_{k}[G(r,\theta )/(r_{0},\theta_{0)}]g(r)F(r,\theta )$

where Λ is the dose rate constant defined as the dose rate per unit air kerma strength at 1 cm along the transverse axis of the source in the water-equivalent medium. Λ (Occu-Prosta) = 0.972 cGy h^−1^ U^−1^

Air kerma strength, S_k_, is a measure of brachytherapy source strength, which is specified in terms of air kerma rate at the point along the transverse axis of the source in free space. Unit of air kerma strength is U. (1U = 1 cGy cm^2^ h^−1^).

The geometry factor G(r, θ), accounts for the variation of relative dose due to the spatial distribution of activity within the source, ignoring photon absorption and scattering in the source structure.

The radial dose function, g (r), accounts for the effects of absorption and scatter in the medium along the transverse axis of the source.

The anisotropy function, F(r, θ), accounts for the anisotropy of dose distribution around the source, including the effects of absorption and scatter in the medium.

r_0_ is the reference distance of 1 cm along the transverse axis of the source.

### Configuration and QA of TPS

It has been recommended to verify single seed calculation results using TPS.[12] In recent years, many reports have been published about treatment planning verification.[12–14] An independent check was undertaken to ensure that the basic input data was entered correctly, which involves source parameters and dosimetric functions.

BrachyVision TPS (Varian Medical Systems, Palo Alto, California) was used for calculation of dose distribution. A unique feature of BrachyVision TPS is that it allows end user to configure different types of radionuclides. The active dimension of the I-125 source, 0.5 mm(Φ) × 3 mm(L), was fed into the system. Once the dimension of the source is known to the system, the sources can be inserted in the planning process by a single click. TPS was configured with all the constants and functions, i.e., dose rate constants, radial dose functions, anisotropy functions, anisotropy factors and geometry functions, using the data from Sharma *et al.*[10] Dose was calculated for water-equivalent tissue based on TG43 formalism.

The dose distribution in transverse axis and off-axis section was calculated. The off-axis measurements and their relationship to the central-axis depth doses were documented. TPS calculation agreed very well with the manual calculation [Table 1].

**Table 1:** Treatment planning system *vs.* manualcalculation dose for a single source

*Polar angle (degree)*	*Radial distance (cm)*	*Dose rate (cGy)*		*Variation (%)*
			
		*TPS*	Manual*	
0	0.5	3.95	4.05	2.5
0	1.0	0.97	0.972	0.2
45	0.5	3.85	3.77	2.1
45	1.0	0.91	0.898	1.3
60	0.5	3.9	3.98	2.01
60	1.0	0.92	0.94	2.1

* Data taken from Ref. 10

### Eye plaque construction

The issues to be considered in design of a plaque are material and thickness of the shield, shape and size of the tumor and required dose distribution. The dimension of plaque was chosen to encompass the tumor volume based on COMS protocol.[15]

**Figure 2:**
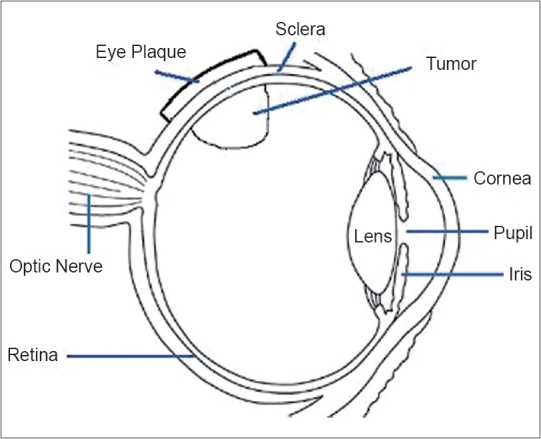
Anatomy of Eye and eye plaque placement

**Figure 3:**
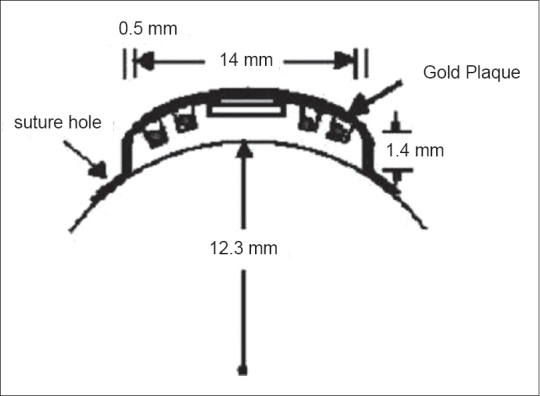
Magnified view of eye plaque placed over scleral surface

Eye plaque was fabricated locally. The plaque was constructed of 17 karat pure gold. The dimension of eye plaque was 15 mm diameter, 12.3 mm radius of curvature and 2 mm thickness. With the HVL of I-125 radiation in pure gold being about 0.01 mm, transmission through the 2-mm gold can be considered to be zero. Plaque was provided with suture holes on the periphery of the plaque. The distance between the surface of the eye and the center of source was considered as 1.4 mm including the radius of seed, which was 0.4 mm; and 1-mm thick adhesive was used to secure the sources. The eye plaque was applied externally to the scleral surface over the tumor base as shown in Figures 2 and 3.

### Dose prescription

The patient in consideration was a male with choroid metastasis in the right eye from carcinoma of colon. After complete evaluation and imaging study, the tumor was found to be 8.6 mm in height and with a basal diameter of 12 mm. Considering tumor-free margin of 1 mm, a plaque of 15-mm diameter was found to be adequate.

For tumors which are 5 mm or greater in apical height, the tumor dose is prescribed at the apex of the tumor; and for tumors which are 2.5-5 mm in apical height, the prescription point is 5 mm from the interior surface of the sclera.[15]

A dose of 60 Gy was prescribed at 10 mm, including the distance of 1.4 mm from the sources to the surface of the eye.

### Radiation source arrangement

The seeds were arranged in an outer ring near the edge of the plaque (within 0.5 mm of the edge) and in concentric rings throughout the plaque. Totally, 12 sources with uniform activity of 2.84U (±5%) were used. The seeds were directly glued to the concave surface of the plaque with cynoacrylate biocompatible adhesive. An additional layer of adhesive was applied over the sources to secure it adequately.

**Figure 4:**
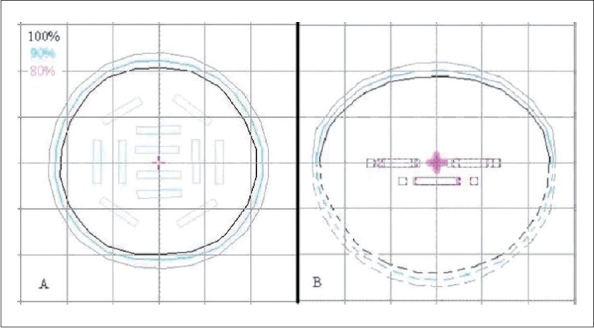
A & B shows dose distribution around the plaque in frontal at 5mm depth & side view

The sources were manually digitized on the TPS by measuring the plaque center-to-seed distance. Dose distribution was calculated in three dimensions Figure 4. To deliver a dose of 60 Gy at 10 mm with 12 sources of total activity of 34.08U, the treatment time calculated was 10 days. The treatment time calculated agreed well within 1.4% of data published by Sharma *et al.* for similar plaque arrangement (Appendix A). Figure 4A shows the dose distribution at 5 mm depth, where 100% isodose line covers 2 cm diameter. The tumor was conical shaped; hence distribution at midplane, i.e., at 5 mm, was evaluated. Figure 4B shows the side view, where 100% isodose line covers apex of the tumor (1-cm depth from plaque).

**Figure 5:**
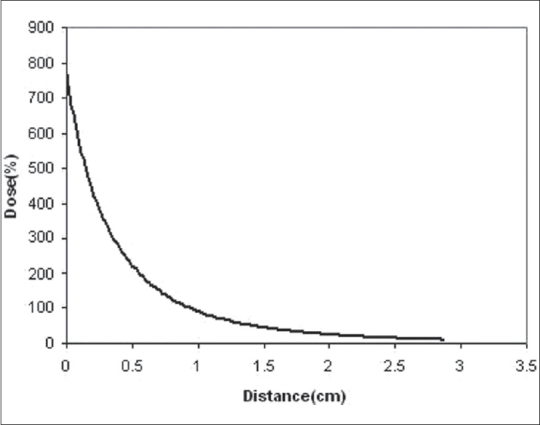
Radial dose fall off on the plaque central axis

The dose falloff from I-125 source was very steep, which is summarized in Figure 5. At 2 cm from the center of eye plaque, dose fell to nearly zero.

## Results and Discussion

The well-type chamber was cross-calibrated against reference well-type chamber having I-125 calibration factor for Amersham model 6711 seed. The calibration factor was found to be 2.68 cGy cm^2^ h^−1^ nA^−1^. Calibration of I-125 sources was done, and results were found to be within ±10% of the activity stated by the manufacturer [Table 2]. Ideally the chamber should be calibrated for Occu-Prosta seed in terms of reference air kerma strength, which was not done in our case. The dependence of calibration factor on different models of I-125 sources is reported to be +15% to −10% relative to Amersham model 6711 seed.[16] However, due to similarity in the construction of Occu-Prosta seed Amersham model 6711, this difference is expected to be not significant.

**Table 2:** Source strength measurement

*Source No.*	*Activity (cGy cm^2^ h^−1^)*		*Variation (%)*
		
	*Quoted (avg.)*	*Measured*	
1	3	3.1	0.33
2	3	3.15	5
3	3	3.3	10
4	3	2.98	−0.7
5	3	3	0
6	3	3.25	8.3

The dosimetric characteristics of low-energy sources, such as I-125, are very sensitive to the details of encapsulation geometry and source internal structure due to self-absorption and filtration effects.[6] Significant dosimetric differences between different seed models containing the same radionuclide may result from relatively minor differences in design specifications or in manufacturing processes. Different constants and functions (i.e., dose rate constants, radial dose functions, anisotropy functions, anisotropy factors and geometry functions) are published in the TG-43 report for Amersham models 6711 and 6702 I-125 sources.[8,17–19] It is inappropriate to use same constants and functions as in TG-43.

The sources were configured into TPS with their 3D coordinates. Based on the input dosimetric data, the 3D dose distribution was calculated. The central axis dose and off-axis dose calculated manually show a good agreement with TPS-calculated dose in homogeneous medium, and it is shown in Table 1.

In our TPS, the effects of adhesive and gold were excluded. Dose-modifying effect of these materials has been analyzed by investigators. Chiu-Tsao *et al.* and Zerda *et al.* concluded that the effect of adhesive and gold plaque was a dose reduction of about 10% at 1 cm on central axis and about 15% at 2 cm at off-axis points.[20,21] The accurate modeling of attenuation due to adhesive, transmission through gold and dose to vicinity of gold had been done by Astrahan.[22] These modifications were accounted for while prescribing the dose. We have assumed dose reduction of about 10% at 1 cm on central axis and accordingly the prescribed dose was increased by 10% with consultation of the oncologist. Our future work will be to configure TPS with these dose modification factors.

## Conclusion

Brachytherapy represents an effective means of treatment for intraocular lesions. BrachyVision TPS can be used for dosimetry of I-125. With availability of I-125 sources locally, cost-effective brachytherapy using eye plaques can be offered in India. Moreover, the I-125 seeds can be explored for treating small nodal volumes, with the help of computerized treatment planning system.

## Appendix A

$\begin{array}{c} \mathrm{Treatment Time}=\frac{\mathrm{Prescribe Dose}(\mathrm{cGy})}{\mathrm{No. of seeds x Activity per seed (U)}}\\ \mathrm{x Dose rate (cGy/h) at 10 mm} \end{array}$

(where dose rate is from ref. 10 for 15 mm plaque with 12 seeds)

$\begin{array}{c} =6000/12x2.840.744\\ =236.63h\\ =\mathrm{9 days and 21 h} \end{array}$
